# Potential biomarkers of miRNA in non-functional pituitary adenomas

**DOI:** 10.1186/s12957-021-02383-3

**Published:** 2021-09-09

**Authors:** Qizhi Zhang, Ying Wang, Yinting Zhou, Qiujuan Zhang, Chuan Xu

**Affiliations:** 1 Department of Neurology, Yueyang Hospital of Integrated Traditional Chinese and Western Medicine, Shanghai University of Traditional Chinese Medicine, No. 110 Ganhe Road, Hongkou District, Shanghai, 200437 China; 2 Department of Ophthalmology, Shanghai Municipal Hospital of Traditional Chinese Medicine, Shanghai University of Traditional Chinese Medicine, Shanghai, 200071 China

**Keywords:** miRNA, Non-functional, Pituitary adenomas, Biomarkers

## Abstract

**Background:**

The abnormal expression of microRNA (miRNA) has been proved to be closely related to the occurrence and progression of tumors. A unique expression of multiple miRNAs has been found in different types of tumors. However, the correlation between miRNA and non-functional pituitary adenoma (NFPA) is not clear. In this study, miRNAs (miRNA-26b, miRNA-138, miRNA-206, and miRNA-let-7e) have been used as detection genes to compare the miRNA expression levels of NFPA subjects and healthy controls and to explore the expression of four different miRNAs in NFPA.

**Methods:**

Ten untreated NFPA volunteers were served as subjects, and 10 normal subjects were selected as controls. Peripheral blood samples were collected, and four differentiated expressed miRNAs (miRNA-26b, miRNA-138, miRNA-206, and miRNA-let-7e) obtained in the early stage of the test group were detected, recorded, and archived by quantitative real-time PCR (qPCR). The difference and significance of endogenous miRNA expressions were explored through statistical analysis, hoping to find biomarkers for clinical treatment.

**Results:**

The levels of miRNA-26b, miRNA-138, miRNA-206, and miRNA-let-7e in the peripheral serum of patients with NFPA were significantly lower than those in normal subjects (*P* < 0.05).

**Conclusion:**

miRNA-26b, miRNA-138, miRNA-206, and miRNA-let-7e may be involved in the occurrence and progress of NFPAs. This study aims to study the biological targets of NFPA. It starts from the study of whether miRNA, miRNA-26b, miRNA-138, miRNA-206, and miRNA-let-7e may be tumor suppressor genes in NFPA, which provides a basis for further exploration of tumor markers of pituitary adenoma.

## Introduction

Pituitary adenoma (PA) is a common intracranial tumor. Epidemiological investigation shows that the incidence is about 15% [1] of all central nervous system tumors, and it is only behind that of glioma and meningioma. With the improvement of diagnostic technology and the popularity of MRI, the rate of case detection has increased with each passing year. According to the level of hormone secretion, they are divided into NFPA and functional PA, including PRL, GH, ACTH, TSH, and LH/FSH. In a narrow sense, NFPA refers to PA without clinical symptoms of excessive secretion of pituitary hormone as well as abnormal increase of serum anterior pituitary hormone level, but with negative electron microscopic observation and immunohistochemical staining. The generalized NFPA also includes the inactive type of various PAs. In this study, clinical NFPAs in a broad sense were discussed. There is no significant gender difference in its incidence, accounting for about 25–35% of PAs [2].

**Fig. 1:**
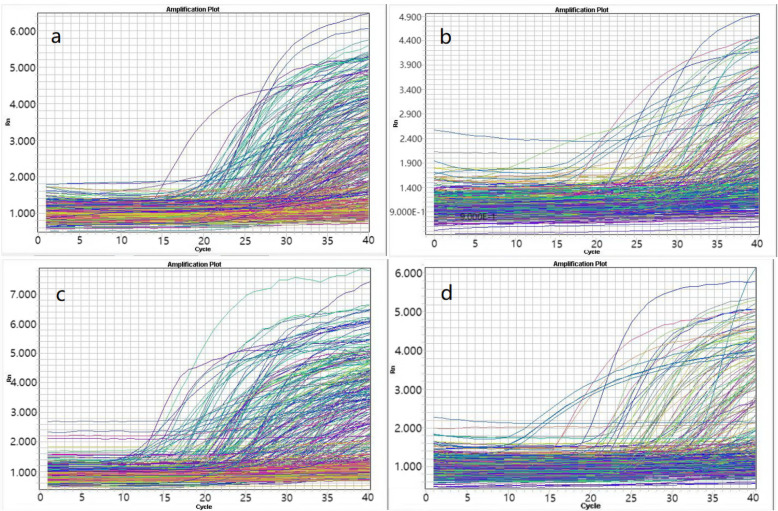
The amplification curve of mi-RNA sample of the serum. **a** Amplification curve of mi-RNA sample of the serum of normal people on the A side of the chip. **b** Amplification curve of mi-RNA sample of the serum of normal people on the B side of the chip. **c** Amplification curve of mi-RNA sample of serum the of patients with hypophysoma of PRL type on the A side of the chip. **d** Amplification curve of mi-RNA sample of the serum of patients with hypophysoma of PRL type on the B side of the chip

In the project “the study on the miRNA expression in PRL pituitary adenoma and Chinese medicine” information intake in 2013, the research group first explored and found the target gene and messenger RNA of endogenous differential miRNA. The research group first detected 20 patients with PRL PA and set 20 healthy people as controls. All miRNAs were extracted and purified. TaqManB human miRNA array set V3.0 low-density chip (A and B boards) was used to detect, including 667 known miRNAs and multiple internal references, a total of 762 miRNAs. Among the 761 miRNAs, 28 miRNAs such as miRNA-let-7e and miRNA-1180 were found to have significant differential expression (Fig. 1). According to the difference in the gene expression between the two groups, the volcanic map was made.

**Fig. 2:**
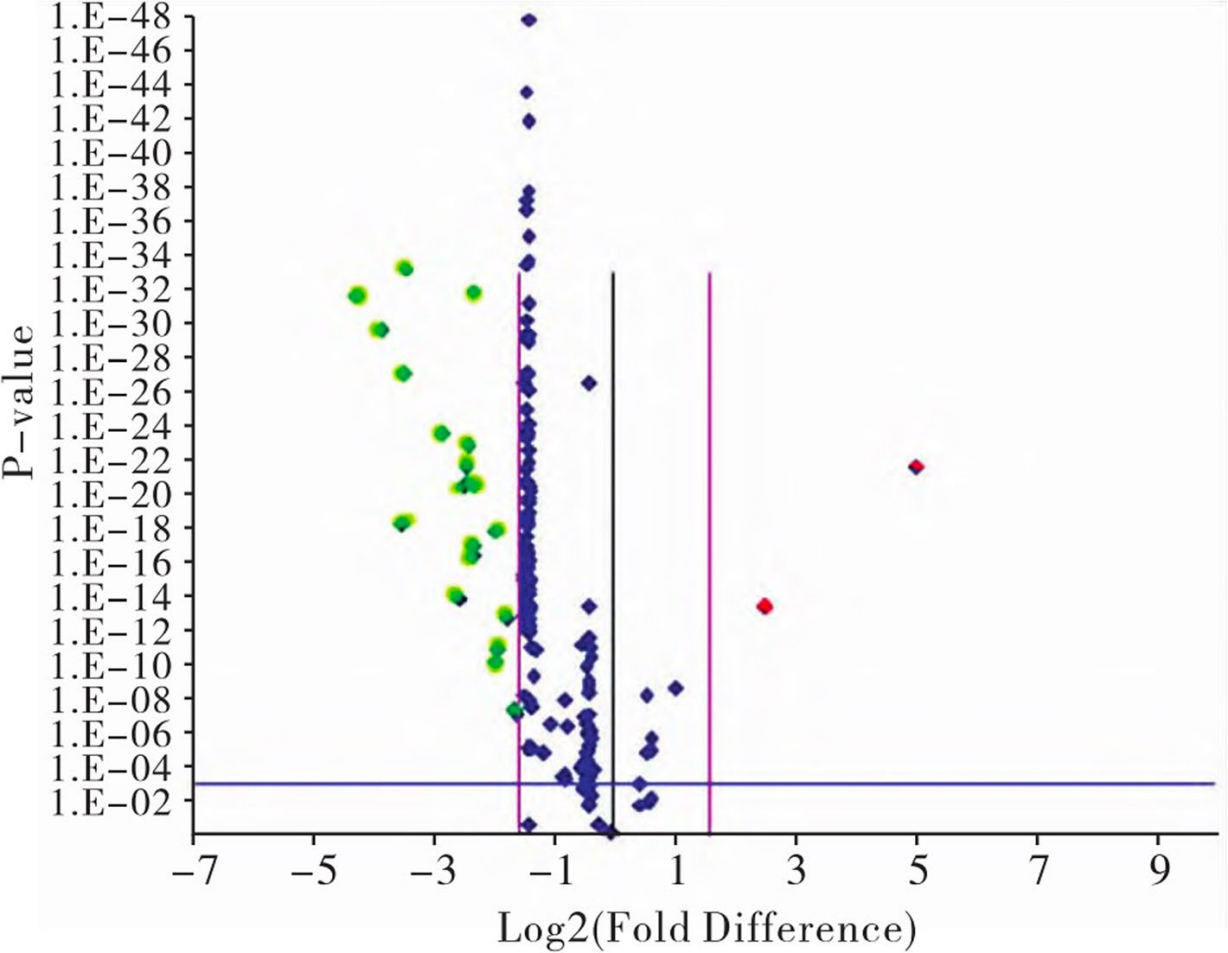
The volcano figure for screening out differentiated mi-RNA

On the map, the *x*-axis represents the log2 conversion value of the mean value of biological repeated expression ratio of the two samples, and the *y*-axis represents the conversion value of −log 10 of the *P*-value value calculated by the *t*-test. Each data point in the figure represents a gene on the chip, and the data points are marked in red or green color on both sides of the crater representing one gene ΔΔCT ≥ 2. These differential multiples combined with a *P*-value ≤ 0.05 are considered to be genes with both differential expression multiples and statistical differences. Blue dots are generally considered to have no significant difference in genes (Fig. 2).

According to the obtained differential miRNAs, six valuable endogenous differential miRNAs, namely miRNA-138, miRNA-1305, miRNA-26b, miRNA-206, miRNA-628-5p, and miRNA-let-7e, were further obtained by cluster analysis, using computer software and related databases and a software called Dateassist. On this basis, 50 cases of PRL PA patients were collected, and their peripheral blood was analyzed by polymerase chain reaction (PCR). The results showed that four miRNAs (miRNA-26b, miRNA-138, miRNA-206, and miRNA-let-7e) were consistent with the results of the first study and were significantly underrated. After these two tests, it can be speculated that four miRNAs mentioned above may also participate in the occurrence and development of other types of PAs, and relevant experimental verification can be arranged accordingly. Based on the above research results, this study will continue to use PCR technology to compare the expression levels of miRNA-26b, miRNA-138, miRNA-206, and miRNA-let-7e between patients with NFPAs and healthy people.

## Material and methods

### Patient

All the samples were from the outpatients of Yueyang Hospital of Integrated Traditional Chinese and Western Medicine, Shanghai University of Traditional Chinese Medicine. This study has passed the ethical review of the ethics committee of Yueyang Hospital of Integrated Traditional Chinese and Western Medicine, Shanghai University of Traditional Chinese Medicine.

Ten patients with NFPA were consistent with the following: (1) dynamic contrast-enhanced MRI in the sellar region showed PA in the sella turcica (space-occupying lesion or abnormal signal in pituitary gland), (2) the abnormal changes of pituitary hormones and their subunits could not be found in laboratory examination, and (3) if the patient had undergone tumor resection, the results of immunocytochemical staining or electron microscopic observation of pituitary tumor tissue were consistent with the pathological diagnosis. The following are the exclusion criteria: (1) patients with other pituitary diseases or abnormal increase or decrease of anterior pituitary hormone level due to physiological or pharmacological reasons; (2) patients with severe primary diseases of the endocrine, circulatory, digestive, reproductive, and blood systems or other solid tumors; (3) pregnant or lactating women; and (4) the patients received radiotherapy and drug treatment before enrollment. Ten healthy people were selected as the control group (there was no abnormality in the dynamic contrast-enhanced MRI of the sellar region). The clinical characteristics of patients were shown in Table 1.

**Table 1:** The clinical characteristics of patients

Order number	Tumor size (mm)	Signal	Relationship between tumor and its surrounding tissue
1	5 × 9	T1WI showed isointense and slight hypointense, T2WI showed slight hyperintense, and the degree of enhancement after dynamic enhancement was weaker than that of normal pituitary.	There was no obvious deviation of the pituitary stalk, and the bone of the sellar floor collapsed slightly. There was no obvious abnormality in the optic chiasm. There was no obvious abnormal signal in the bilateral cavernous sinus.
2	6 × 8	T1WI showed isointense and hypointense, T2WI showed isointense, and dynamic enhancement showed enhancement and less uniform, weaker than normal pituitary enhancement.	The pituitary stalk shifted to the right, and the bone of the sellar floor collapsed slightly. The left cavernous sinus was involved.
3	20 × 13	Patchy short T1, slightly long T2 signal, and enhanced lesions showed mild enhancement.	The pituitary stalk is not clearly displayed. There was no abnormal signal in the bilateral cavernous sinus, and the optic chiasm was slightly compressed and raised.
4	5.8 × 7	T1WI showed slightly low signal, T2WI showed uneven slightly high signal, and the lesions showed mild enhancement after enhancement.	The pituitary stalk was in the middle, no obvious elevation of the dorsal sella, no obvious collapse of the sellar floor, and no obvious abnormal enhancement of the bilateral cavernous sinus structure.
5	38 × 30	The signal intensity was similar to that of white matter and enhanced significantly after enhancement.	There was a gourd-shaped isosignal shadow in the pituitary area with a clear boundary and no abnormal widening of the ventricular system. No displacement of midline structure.
6	25 × 30	T1WI showed isointense and slightly hypointense, T2WI showed mixed hyperintense, cystic changes were seen in some lesions, and the parenchyma showed obvious homogeneous enhancement after dynamic enhancement.	The pituitary stalk shifted to the right, and the bone of the sellar floor collapsed. The visual chiasm is not clear. The boundary between the lesion and bilateral cavernous sinus was not clear.
7	3.3 × 8.3	The boundary was clear, isointense and hypointense on T1WI, isointense on T2WI, and enhanced after dynamic enhancement, which was less homogeneous than normal pituitary.	The pituitary stalk shifted to the right, and the bone of the sellar floor did not collapse.
8	33 × 28	T1WI showed equal low mixed signal, T2WI showed equal high mixed signal, and enhanced scan showed uneven enhancement.	The tumor invaded the bilateral cavernous sinus, protruded to the parasellar and suprasellar, extended into the suprasellar cistern and sphenoid sinus, destroyed the bone at the sellar floor, and compressed the bilateral optic chiasm.
9	13 × 14	On the contrast-enhanced scan, the lesions showed relatively low signal intensity.	Partial filling defect of the left cavernous sinus, mild enlargement of the sella turcica, partial loss of the anterior and inferior bone, and mild right deviation of the pituitary stalk.
10	12.3 × 14.1	T1WI sequence showed edge isointensity and internal patchy hyperintensity. T2WI sequence showed mixed isointensity. After contrast enhancement, the lesions were peripheral enhancement, and the middle part of the hyperintensity had no obvious enhancement, and the signal was uneven.	The sella turcica was enlarged, the bone of the sellar floor was slightly compressed, and the optic chiasm was compressed and moved up.

### Experimental methods

Two milliliters of peripheral blood was collected by an ethylene diamine tetraacetic acid (EDTA) vacuum vessel. After the blood sample was collected, it was centrifuged at 3500 rpm/min by ordinary centrifuge at room temperature. After centrifugation, the upper serum was collected and mixed with the denatured solution of equal volume. (a) The lysate for RNA isolation was transferred to a test tube containing an equivalent amount of 2× denatured solution at room temperature and (b) immediately mixed thoroughly and incubated on ice for 5 min. Another volume of acidic phenol was mixed with it by eddy current for 30–60 s, centrifuged at the maximum speed (≥ 10,000×*g*) at room temperature for 5 min, and the supernatant was removed and transferred to a new tube; 1.25 volume of 100% ethanol was added at room temperature for total RNA isolation. After centrifugation for about 30 s, the RNA was recovered and centrifuged briefly. The TaqMan miRNA reverse transcription kit was used for reverse transcription for 65 min. The main mixture of RT reaction was prepared, and qPCR amplification was performed for 120 min. The reagents were thawed and mixed. The number of reactions required for each analysis was calculated and recorded in the PCR system software. Finally, the calculated results were compared with the standard curve to obtain the expression level of differential miRNA.

### Data analysis

The statistical data were processed by the SPSS 22.0 software. The Fisher exact probability method was used to compare the general data. The results of age and qPCR (expressed by ΔCT) were statistically described by two independent samples *t*-test and ± *s*; those not conforming to normal distribution or homogeneity of variance were statistically described by non-parametric test and *m* (min, max). Both sides were tested, with *P* < 0.05 statistically significant.

## Results

### General information analysis

In this study, there were 2 males and 8 females, with an average age of 47.7, as shown in Table 2; in the control group, there were 5 males and 5 females, with an average age of 46.2, as shown in Table 3. In the process, the Fisher exact probability was used. After testing, the difference was not statistically significant (*P* > 0.05), and the data were comparable.

**Table 2:** Comparison of subjects and healthy people

Group	Gender		
	Case (*n*)	Male	Female
Subjects	10	2	8
Healthy people	10	5	5
*P* value	0.350

**Table 3:** Age comparison between subjects and healthy people

Group	Age					Average age
	Case (*n*)	20–34	35–49	50–64	65–79	
Subjects	10	1	4	5	0	47.7
Healthy people	10	4	2	2	2	46.2
*P* value	0.846					

### Comparison of miRNA ΔCT levels between NFPA subjects and healthy subjects

The qPCR data of miRNA-26b met the requirements of normality and homogeneity of variance. The contrast level of miRNA ΔCT of the two groups was expressed by ± *s* (three decimal places are reserved, the same below): miRNA-26b ΔCT level contrast, *t* = 4.751, *P* < 0.001. Because the qPCR data of miRNA-138, miRNA-206, and miRNA-let-7e met the normality and homogeneity of variance at different times, the non-parametric test was implemented. Because the disease is rare, the total number of samples is small, which is expressed by *m* (min, max): miRNA-138 ΔCT level contrast, *z* = − 2.948, *P* = 0.003; miRNA-206 ΔCT level contrast, *z* = − 3.250, *P* < 0.001; miRNA-let-7e level comparison, *z* = − 3.780, *P* < 0.001. The results showed that the expression levels of miRNA-138, miRNA-26b, miRNA-206, and miRNA-let-7e were significantly lower than those of healthy controls (*P* < 0.05). Detailed data are shown in Tables 4, 5, 6, and 7.

**Table 4:** Comparison of miR-138 ΔCT levels between subjects and healthy people: *m* (min, max)

Group	Case (*n*)	*m* (min, max)	
Subject	10	19.454 (16.023, 27.355)	*Z* = − 2.948
Healthy people	10		*P* = 0.003

**Table 5:** Comparison of miR-206 ΔCT levels between subjects and healthy people: *m* (min, max)

Group	Case (*n*)	*m* (min, max)	
Subject	10	23.023 (18.827, 28.287)	*Z* = − 3.250
Healthy people	10		*P* < 0.001

**Table 6:** Comparison of miR-26b ΔCT levels between subjects and healthy people: ($\overline{x}\pm s$)

Group	Case (*n*)	Mean differences${}^{\overline{x}}$	Standard deviation (*s*)	
Subject	10	15.503	1.858	*t* = 4.751
Healthy people	10	20.493	2.753	*P* < 0.001

**Table 7:** Comparison of miR-let-7e ΔCT levels between subjects and healthy people: ($\overline{x}\pm s$)

Group	Case (*n*)	*m* (min, max)	
Subject	10	25.725 (16.980, 33.950)	*Z* = − 3.780
Healthy people	10		*P* < 0.001

## Discussion

At present, the pathogenesis of PA mainly includes the following theories: oncogene theory [3], tumor suppressor gene theory [4], epigenetic change theory [5], hypothalamic out of control regulation theory [6], pituitary cell self-defect theory [7], hormone regulation theory [8], and miRNA imbalance and cytokine and growth factor imbalance theory [9, 10], etc. However, due to the defects of the existing theories, there is still no authoritative conclusion, and many disputes still exist. In recent years, the research on miRNA is in the stage of vigorous development. The abnormal expression of miRNA has been proved to be closely related to the occurrence and development of a variety of tumors, and their unique characteristic expressions have been found in different types of tumors, which have different regulatory mechanisms for the development and prognosis of tumors. Mir-29a can specifically inhibit the proliferation of liver cancer cells [11]. Mir-10b-3p can promote the proliferation, invasion, and migration of HCC cells [12]. Some studies [13, 14] have found that the Mir-149 gene may be associated with the increased risk of gastric cancer in Asians, while other studies have suggested that Mir-149 can enhance the cisplatin sensitivity of EC cell lines and inhibit the development of esophageal cancer.

Through the transfection analysis of hepatocytes with different metastatic potential [15], it is found that the overexpression of mirRNA-26b can obviously inhibit the proliferation and metastasis of hepatocellular carcinoma, which provides a new point of view for the treatment of hepatocellular carcinoma. Wang et al. [16] found that Jag1, as a target of miRNA-26b, has a negative correlation with it, suggesting that the miRNA-26b/Jag1 axis may become a new therapeutic target.

In the study of Manafi Shabestari et al. [17], miRNA-138 has been confirmed as a tumor suppressor gene. In the telomerase activity and cell proliferation of acute promyelocytic leukemia NB4 cells, it has been discovered that miRNA-138 can induce apoptosis of malignant tumor cells through the mediation of caspase. In the study of You et al. [18], it has come under observation that miRNA-138, as a vector, is often lowly expressed in colon cancer tissue. It is closely related to the proliferation, metastasis, and prognosis of colon cancer cells. In the experiment, it has been found that miRNA-138 is negatively correlated with the malignant degree of the tumor but is positively correlated with the prognosis and survival rate of patients.

Mirna-206 has a protective effect on colon cancer induced by prostaglandin E2 [19]. At the miRNA and protein levels, the expression of miRNA-206 significantly reduces the expression of transmembrane-4l-six family member-1 (tm4sf1) in promoting the proliferation and migration of colorectal cancer cells. Quan et al. [20] have found that different from the role of tumor suppressor gene of miRNA-206 in colon cancer and prostate cancer, miRNA-206 has obvious high expression in breast cancer tissue. As an oncogene in breast cancer, miRNA-206 may be closely related to the prognosis of patients.

The miRNA-let-7 family has the function of both supressor of tumor and oncogene and an extremely important differentiation regulator [21, 22]. Han et al. [23] through the study of the role of miRNA-let-7 in the chemosensitivity of gastric cancer patients to cisplatin have found that miRNA-let-7 simulation transfection can significantly reduce the growth of primary tumor and provide a new treatment for reducing the cisplatin resistance of gastric cancer cells. In another study, for the migration and invasion of tongue squamous cell carcinoma (TSCC), long non-coding RNA H19 is highly expressed in TSCC cells, which has the ability to promote the migration and invasion of tumor cells, while H19-mediated invasion of TSCC promotes tumor metastasis in a let-7e-dependent manner through the epithelial-mesenchymal transition (EMT) pathway [24]. The research on the miRNA-let-7 family is still expanding.

In the study of the effect of miRNA on pituitary tumor, it has been found that its role includes local expression and circulating form. Objective to identify potential biomarkers by detecting circulating miRNA and isomiR levels in plasma samples of patients with pituitary adenomas before and after surgery, next-generation sequencing (NGS) has been used to compare the plasma samples before and after transsphenoidal pituitary surgery with those of normal people. It has been concluded that the number of miRNA reading in the experimental group is significantly lower than that in the control group. It has shown significant differences in the expression of mature miRNA and isomiR between the experimental group and the control group, indicating the expression of miRNA-143-3p is significantly higher in FSH/LH1 pituitary adenoma patients by qPCR, and it is significantly lower after surgery [25].

Clinical NFPAs usually lack early clinical symptoms when the tumor is small (< 10 mm) because they have no active hormone secretion. Most of the tumors have been found occasionally during physical examination due to other systemic symptoms. There are cavernous sinus and carotid artery on both sides of the pituitary gland, and there is optic chiasm on them. In view of the special position of the pituitary gland, when the tumor increases and grows upward, the visual field defect will appear when the tumor oppresses the optic chiasm, and a few patients will have ophthalmoplegia. When the tumor expands to the surrounding brain tissue, according to the different compression positions, the patients will have atypical symptoms in the corresponding brain functional areas, such as frontal lobe behavioral changes with cognitive impairment and temporal lobe dementia [26–28]. When the size of the tumor is large and tumor-occupying effect occurs, dizziness, headache, nausea and vomiting, and other symptoms may occur due to the disturbance of cerebrospinal fluid circulation, or pituitary dysfunction may be caused by compression of normal pituitary tissue [29].

The commonly used Western medicine treatment methods for clinical NFPA include surgery, radiotherapy, and drugs. At present, surgery is the mainstream treatment, because surgery can appropriately reduce the symptoms of intracranial local compression, to a certain extent, and alleviate the optic chiasmatic involvement. Statistics showed that after the occurrence of obvious compression symptoms, the curative effect of surgical intervention is significantly improved, and the adverse reactions are significantly reduced [30]. At present, radiotherapy is the second-line adjuvant therapy. Some researchers have found that there are dopamine receptor and somatostatin receptor on the cell membrane of non-functional adenoma, so it has certain curative effects to use dopamine receptor agonist (DA), somatostatin analogs (SSA), and their chimeric ligands [31, 32].

## Conclusion

In this paper, we have mainly studied the targets of NFPA. Only 10 cases with normal pituitary hormone levels and 10 persons with normal serum pituitary hormone levels and negative dynamic contrast-enhanced MRI in the sellar region have been observed. It has been found that miRNA-138, miRNA-206, miRNA-26b, and miRNA-let-7e have been underrated in the serum of patients with NFPA. It can be speculated that these four genes may be new biological targets for the treatment of NFPA, providing the basis for the treatment of NFPA in the future. Because this disease is rare, only 10 cases have been studied in this study, and the number of cases observed is small. Due to the lack of previous studies, this observation is limited to the abnormal expression of four differential miRNAs in patients with non-functional pituitary adenoma. In the future study, we can further observe the correlation between miRNA expression and tumor size, tumor, and surrounding tissues.

## Data Availability

The datasets used and/or analyzed during the current study are available from the corresponding author on reasonable request.

## References

[1] Theodros D, Patel M, Ruzevick J, Lim M, Bettegowda C (2015). Pituitary adenomas: historical perspective, surgical management and future directions. CNS Onco1.

[2] Colao A, Di Somma C, Pivonello R, Faggiano A, Lombardi G, Savastano S (2008). Medical therapy for clinically non-fuctioning pituitary adenoma. Endorinol Relat Cancer..

[3] Auriemma RS, Pivonello R, Ferreri L, Priscitelli P, Colao A (2015). Cabergoline use for pituitary tumors and valvular disorders. Endocrinol Metab Clin North Am..

[4] Liang M, Chert X, Liu W (2011). Role of the pituitary tumor transforming gene l in the progression of hepatocellular carcinoma. Cancer Biol Ther..

[5] Pease M, Ling C, Mack WJ, Wang K, Zada G (2013). The role of epigenetic modification in tumorigenesis and progression of pituitary adenomas: a systematic review of the literature. PLoS One..

[6] Davis SW, Potok MA, Brinkmeier ML, Carninci P, Lyons RH, MacDonald JW, Fleming MT, Mortensen AH, Egashira N, Ghosh D, Steel KP, Osamura RY, Hayashizaki Y, Camper SA (2009). Genetics, gene expression and bioinformatics of the pituitary gland. Horm Res..

[7] Vandeva S, Tichomirowa MA, Zacharieva S, Daly AF, Beckers A (2010). Genetie factors in the development of pituitary adenomas. Endocr Dev..

[8] Strathmann FG, Borlee G, Born DE, Gonzalez-Cuyar LF, Huber BR, Baird GS (2012). Multiplex immunoassays of peptide hormones extracted from formalin-fixed, paraffin-embedded tissue accurately subclassify pituitary adenomas. Clin Chem..

[9] Sapochnik M, Nieto LE, Fuertes M, Arzt E (2016). Molecular mechanisms underlying pituitary pathogenesis. Biochem Genet..

[10] Melmed S (2011). Pathogenesis of pituitary tumors. Nat Rev Endocrinol..

[11] Zhang Y, Yang L, Wang S, Liu Z, Xiu M (2018). miR-29a suppresses cell proliferation by targeting SIRT1 in hepatocellular carcinoma. Cancer Biomark..

[12] Guan L, Ji D, Liang N, Li S, Sun B (2018). Up-regulation of miR-10b-3p promotes the progression of hepatocellular carcinoma cells via targeting CMTM5. Cell Mol Med..

[13] Zhang L, Liu Q, Wang F (2018). Association between miR-149 gene rs2292832 polymorphism and risk of gastric cancer. Arch Med Res..

[14] Wang Y, Chen J, Zhang M, Zhang W, Li M, Zang W, Dong Z, Zhao G (2018). miR-149 sensitizes esophageal cancer cell lines to cisplatin by targeting DNA polymerase beta. Cell Mol Med..

[15] Lin CZ, Ou RW, Hu YH (2018). Lentiviral-mediated microRNA-26b up-regulation inhibits proliferation and migration of hepatocellular carcinoma cells. Kaohsiung J Med Sci..

[16] Wang L, Wang W, Wu Y (2019). MicroRNA-26b acts as an antioncogene and prognostic factor in cervical cancer. Oncol Lett..

[17] Manafi Shabestari R, Alikarami F, Bashash D, Paridar M, Safa M (2018). Overexpression of MiR-138 inhibits cell growth and induces caspase-mediated apoptosis in acute promyelocytic leukemia cell line. Int J Mol Cell Med..

[18] You C, Jin L, Xu Q, Shen B, Jiao X, Huang X (2019). Expression of miR-21 and miR-138 in colon cancer and its effect on cell proliferation and prognosis. Oncol Lett..

[19] Park YR, Seo SY (2018). Kim SL, et al MiRNA-206 suppresses PGE2-induced colorectal cancer cell proliferation, migration, and invasion by targetting TM4SF1. Biosci Rep..

[20] Quan Y, Huang X, Quan X (2018). Expression of miRNA-206 and miRNA-145 in breast cancer and correlation with prognosis. Oncol Lett..

[21] Copley MR, Babovic S, Benz C, Knapp DJHF, Beer PA, Kent DG, Wohrer S, Treloar DQ, Day C, Rowe K, Mader H, Kuchenbauer F, Humphries RK, Eaves CJ (2013). The Lin28b-let-7-Hmga2 axis determines the higher self-renewal potential of fetal haematopoietic stem cells. Nat Cell Biol..

[22] Jayaraman M, Radhakrishnan R, Mathews CA, Yan M, Husain S, Moxley KM, Song YS, Dhanasekaran DN (2017). Identification of novel diagnostic and prognostic miRNA signatures in endometrial cancer. Genes Cancer..

[23] Han X, Zhang JJ, Han ZQ, Zhang HB, Wang ZA (2018). Let-7b attenuates cisplatin resistance and tumor growth in gastric cancer by targeting AURKB. Cancer Gene Ther..

[24] Kou N, Liu S, Li X, Li W, Zhong W, Gui L, Chai S, Ren X, Na R, Zeng T, Liu H (2019). H19 facilitates tongue squamous cell carcinoma migration and invasion via sponging miR-let-7. Oncol Res..

[25] Németh K, Darvasi O, Likó I, Szücs N, Czirják S, Reiniger L, Szabó B, Krokker L, Pállinger É, Igaz P, Patócs A, Butz H (2019). Comprehensive analysis of circulating microRNAs in plasma of patients with pituitary adenomas. J Clin Endocrinol Metab..

[26] Daly AF, Beckers A (2020). The epidemiology of pituitary adenomas. Endocrinol Metab Clin North Am..

[27] Deepak D, Daousi C, Javadpour M, MacFarlane IA (2007). Macroprolactinomas and epilepsy. Clin Endocrinol (Oxf)..

[28] Ilovayskaya IA (2018). Dreval’ AV, Krivosheeva YG, Glazkov AA, Astaf’eva LI, Stashuk GA. Clinical and functional characteristics of giant pituitary adenomas in the population of patients in the Moscow region. Zh Vopr Neirokhir Im N N Burdenko..

[29] Ntali G, Wass JA (2018). Epidemiology, clinical presentation and diagnosis of non-functioning pituitary adenomas. Pituitary..

[30] Casanueva FF, Barkan AL, Buchfelder M (2017). Criteria for the definition of Pituitary Tumor Centers of Excellence (PTCOE): a pituitary society statement. Pituitary..

[31] Greenman Y, Cooper O, Yaish I, Robenshtok E, Sagiv N, Jonas-Kimchi T, Yuan X, Gertych A, Shimon I, Ram Z, Melmed S, Stern N (2016). 2016 Treatment of clinically nonfunctioning pituitary adenomas with dopamine agonists. Eur J Endocrinol..

[32] Batista RL, Musolino NRC, Cescato VAS, da Silva GO, Medeiros RSS, Herkenhoff CGB, Trarbach EB, Cunha-Neto MB (2019). Trarbach EB & Cunha-Neto MB 2019 Cabergoline in the management of residual nonfunctioning pituitary adenoma: a single-center, open-label, 2-year randomized clinical trial. Am J Clin Oncol..

